# The efficacy and safety of sacituzumab govitecan in the treatment of breast cancer: a systemic review and meta-analysis of emerging clinical data

**DOI:** 10.3389/fimmu.2025.1683594

**Published:** 2025-11-06

**Authors:** Lili Jiang, Youran Dai, Man Li, Zuowei Zhao

**Affiliations:** 1 Department of Breast Surgery and Department of Oncology, The Second Affiliated Hospital of Dalian Medical University, Dalian, China; 2 Department of Traditional Chinese Medicine, Beijing Shijitan Hospital Affiliated to Capital Medical University, Beijing, China

**Keywords:** Sacituzumab govitecan, breast cancer, meta-analysis, antibody-drug conjugate, systemic review

## Abstract

**Introduction:**

Sacituzumab govitecan (SG) as an antibody-drug conjugate targeting Trophoblast cell surface antigen 2, has emerged as a promising therapy for breast cancer. However, the efficacy of SG across disease subtypes, treatment settings, and in combination regimens remains incompletely defined.

**Materials and methods:**

A comprehensive literature search was conducted in PubMed, Embase, Web of Science, and the Cochrane Library to identify studies reporting the clinical efficacy and safety outcomes of SG in breast cancer. Pooled analyses were performed for overall response rate (ORR), progression-free survival (PFS), overall survival (OS), and treatment-related adverse events (AEs). Subgroup analyses were performed by molecular subtype, disease stage, and treatment regimen.

**Results:**

A total of 13 studies involving 2,447 patients with breast cancer were included. SG significantly improved ORR (OR = 3.97, 95%CIs: 1.32-11.90) and OS (HR = 0.59, 95%CIs: 0.47-0.75) versus single agent chemotherapy in RCTs, with pronounced benefit in metastatic triple-negative breast cancer (mTNBC) (ORR = 10.55; HR for OS: 0.50, 95%CIs: 0.43-0.58). Pooled median PFS (mPFS) was 4.95 months (95%CIs: 4.36-5.61months) in RCTs and 5.93 months (95%CIs: 4.76-7.39 months) in single-arm studies, with early-stage TNBC achieving mPFS up to 9.50 months (95%CIs: 8.91-10.13 months). Combination with immunotherapy suggested numerically longer survival (median OS 18.0 *vs* 12.2 months). The most frequent grade ≥3 AE was neutropenia, occurring in 26-57% of patients, with overall toxicity manageable and consistent across studies.

**Conclusions:**

SG provides substantial clinical benefit in breast cancer, improving ORR, OS, and PFS, particularly in TNBC, with consistent efficacy across monotherapy and combination regimens. The increased risk of hematologic and gastrointestinal toxicities warrants careful monitoring in clinical practice.

**Systematic review registration:**

https://www.crd.york.ac.uk/PROSPERO/, identifier CRD420251072321.

## 1 Introduction

According to the GLOBOCAN 2022 estimates (updated in 2024), breast cancer remains the most frequently diagnosed malignancy among women worldwide, accounting for approximately 23.8% of all new female cancer cases. In 2022, there were an estimated 2.4 million new cases of breast cancer globally, accompanied by 685,000 deaths (1). The highest incidence rates were observed in high-income countries such as the United States and western Europe, while mortality rates in these regions remain comparatively low. In contrast, low- and middle-income countries such as India, bear a disproportionately high mortality burden, reflecting persistent disparities in access to early diagnosis, systemic therapy, and overall cancer care (2).

Breast cancer is a biologically heterogeneous disease encompassing multiple subtypes with distinct molecular profiles and treatment responses (3). Despite significant therapeutic advances, systemic treatment remains challenging, especially in aggressive subtypes and metastatic breast cancer (mBC) (4). Conventional cytotoxic chemotherapy has long served as a cornerstone of breast cancer treatment, particularly in triple-negative and advanced-stage. However, the non-selective mechanism of action often leads to substantial off-target toxicity, limiting both the tolerability and long-term efficacy (5). In addition, resistance to chemotherapeutic agents is frequently observed in clinical practice, further compromising treatment outcomes (6). The advent of monoclonal antibodies (mAbs) has enhanced the specificity of cancer therapies and partially expanded available treatment options (7). Nevertheless, the intrinsic cytotoxicity of mAbs is relatively limited, and they often fail to induce sustained tumor regression when used alone, particularly in rapidly proliferating or drug-resistant tumors (8). Antibody-drug conjugates (ADCs), which link tumor-specific mAbs to highly potent cytotoxic agents via specialized linkers, represent a promising therapeutic strategy that combines targeted delivery with effective tumor cell killing (9). By improving the therapeutic index and reducing systemic toxicity, ADCs have emerged as a key component of precision oncology.

Trophoblast cell surface antigen 2 (Trop-2) is a transmembrane glycoprotein overexpressed in various epithelial malignancies, including breast cancer (10), lung cancer (11), and urothelial carcinoma (12). Notably, Trop-2 is highly expressed in over 80% of triple-negative breast cancers (TNBC) (13) and is associated with enhanced tumor proliferation, invasion, metastasis, and poor prognosis, making it an attractive target for ADCs development (14). These insights led to the development of sacituzumab govitecan (SG), a first-in-class Trop-2-directed ADC designed to address the therapeutic void in metastatic TNBC (mTNBC). SG consists of a humanized anti-Trop-2 monoclonal antibody (hRS7 IgG1κ) conjugated via a hydrolyzable CL2A linker to SN-38, the active metabolite of irinotecan and a potent topoisomerase I inhibitor. Unlike other conventional ADCs that rely solely on internalization into antigen-expressing cells, SG is engineered to release SN-38 both intracellularly and into the tumor microenvironment, enabling a bystander effect that enhances anti-tumor activity while minimizing off-target toxicity (15).

In 2020, the U.S. Food and Drug Administration (FDA) granted accelerated approval to SG for the treatment of mTNBC patients who had received ≥2 prior systemic therapies (16). Since then, multiple clinical trials have demonstrated that SG, as the first Trop-2-targeted ADC approved for breast cancer, can significantly improve outcomes in heavily pretreated patients. Moreover, emerging evidence suggests SG may also provide clinical benefit in hormone receptor-positive/HER2-negative (HR+/HER2-) metastatic breast cancer, expanding its potential application across molecular subtypes and reinforcing the role in precision therapy. Despite these promising findings including improvements in overall response rate (ORR), progression-free survival (PFS), and overall survival (OS), questions remain regarding the consistency of treatment efficacy across subpopulations, the spectrum of treatment-related adverse events, and the generalizability of results to broader clinical settings. Given the recent regulatory approval and limited real-world experience, further investigation is warranted.

In this meta-analysis, we aim to comprehensively evaluate the efficacy and safety of SG in both TNBC and HR+/HER2- breast cancer, based on data from real word. We provide a rigorous comparative synthesis of direct and indirect evidence to inform clinical decision-making and future research directions.

## 2 Materials and methods

This systematic review and meta-analysis were conducted in accordance with the recommendations outlined in the Cochrane Handbook for Systematic Reviews of Interventions and adhered to the Preferred Reporting Items for Systematic Reviews and Meta-Analyses (PRISMA) 2020 guidelines (17, 18). The protocol for this study was registered in PROSPERO (registration number: CRD420251072321).

### 2.1 Search strategy

A comprehensive search was performed in PubMed, Embase, Cochrane Library, and Web of Science, as well as in the abstract archives of major oncology conferences, including those from the European Society for Medical Oncology (ESMO) and the American Society of Clinical Oncology (ASCO). The search was limited to studies published in English up to June 2025, using the following search terms: (“Breast Cancer” OR “Breast Neoplasms”) AND (“Sacituzumab Govitecan”). A detailed search strategy is provided in **Supplementary Table 1**. We also screened reference lists of the included articles, relevant reviews, prior meta-analyses, and unpublished trials to identify additional eligible studies. In the case of multiple publications from the same clinical trial, the most recent and/or complete report was used for data extraction.

### 2.2 Eligibility criteria

Studies were included based on the following predefined criteria: a) original articles reporting prospective or retrospective clinical trials or observational cohort studies, b) enrolled patients diagnosed with breast cancer, c) evaluated SG as monotherapy or part of combination therapy, with SG as the primary intervention, d) reported at least one efficacy outcome (e.g., ORR, PFS, OS) or treatment-related adverse events, e) published in English. The following exclusion criteria were applied: a) non-original publications (e.g., reviews, editorials, commentaries, case reports, animal studies, or study protocols), b) lacked relevant clinical outcome data; c) involved duplicate or overlapping patient cohorts.

### 2.3 Quality assessment

The risk of methodological bias was assessed using two validated tools: the Revised Cochrane Risk of Bias tool (19) for randomized controlled trial (RCTs, RoB 2.0, Version 2), and the Risk of Bias in Non-randomized Studies of Interventions (ROBINS-I) tool (20) for single-arm studies. Two independent reviewers (LJ and YD) conducted the risk-of-bias assessment for each included study. Any discrepancies or disagreements were resolved through discussion with a third reviewer, who provided adjudication and methodological oversight. The Egger test and funnel plots of individual study weights against point estimates were used to verify publication bias for the primary outcome.

### 2.4 Data extraction and management

Two authors (LJ and YD) independently performed data extraction using a standardized extraction form. Key information collected from each included study comprised the following: first author, country, year of publication, trial name, trial phase, study design, sample size, treatment arms, molecular subtype, cancer stage, patients’ age, median follow-up duration, and reported clinical endpoints. Any discrepancies between reviewers were resolved through discussion with a third author during the data extraction process.

### 2.5 Statistical analysis

All statistical analyses were performed using RStudio (version 4.4.2) and Stata 17.0 (StataCorp, College Station, TX, USA). For RCTs, we analyzed dichotomous outcomes including ORR and adverse events (AEs) using odds ratios (ORs) with 95% confidence intervals (CIs). Time-to-event outcomes including PFS and OS were analyzed using hazard ratios (HRs) with 95% CIs. Additionally, we calculated pooled results for ORR, AEs incidence, median PFS and median OS in the experimental arms. For single-arm studies, we computed proportions with 95% CIs for ORR and AES, and summarized reported median PFS and OS times. Subgroup analyses were conducted based on treatment regimens and tumor types. Statistical heterogeneity was assessed using Cochran’s Q χ² test and I² statistics, with P<0.05 for the Q test or I²>50% indicating significant heterogeneity. Given the clinical heterogeneity arising from varying treatment protocols and patient characteristics, we employed random-effects models (DerSimonian-Laird method) for all meta-analyses to enhance result reliability. Publication bias was evaluated using Begg’s test and funnel plots. Sensitivity analyses were performed by sequentially excluding individual studies to verify result stability. A two-sided P-value <0.05 was considered statistically significant for all analyses.

## 3 Results

### 3.1 Search results

**Figure 1:**
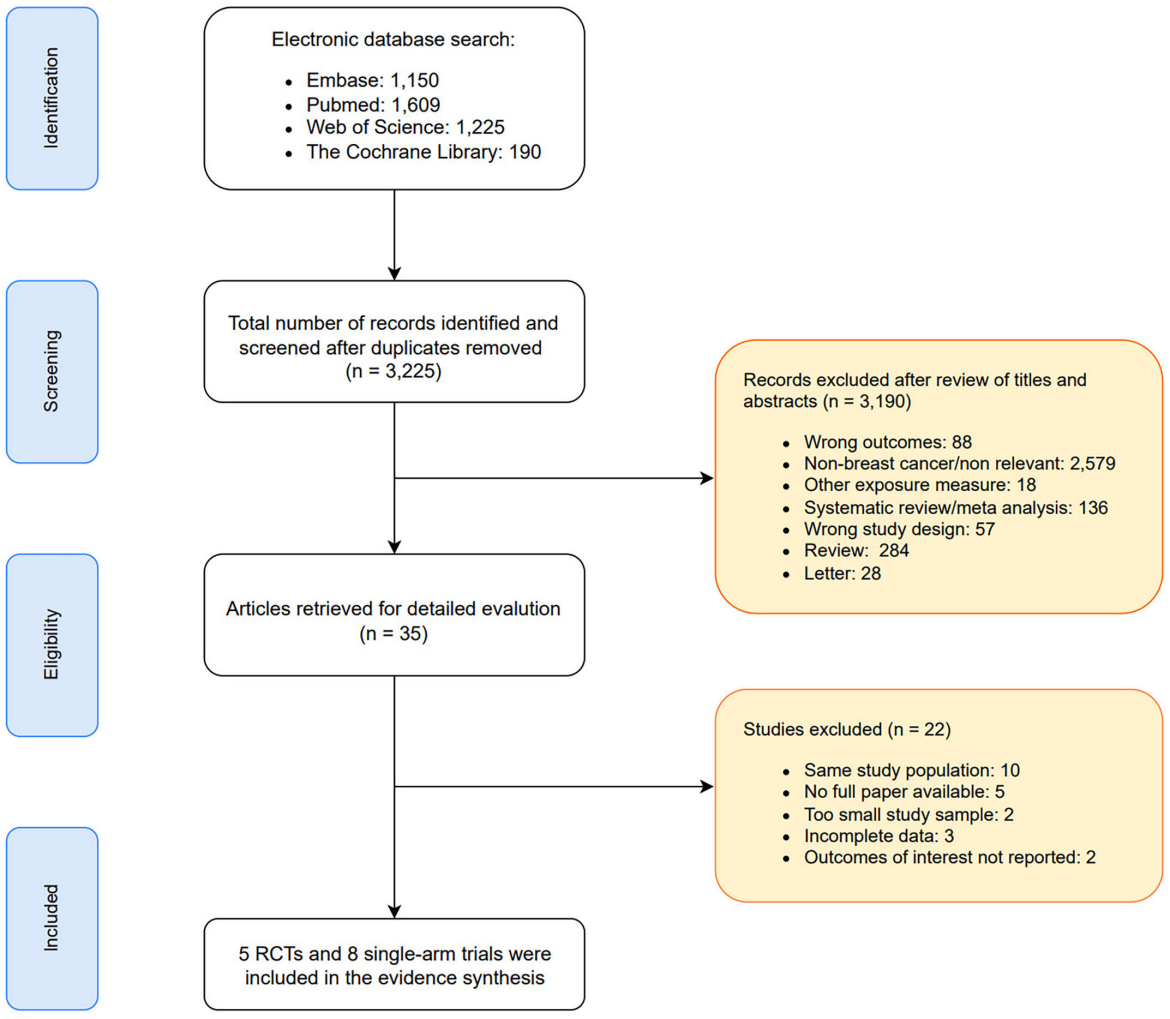
PRISMA diagram of identifying eligible studies.

As depicted in the PRISMA flow diagram (**Figure 1**), a total of 4,174 records were initially retrieved through database searching. After removing 949 duplicates, 3,225 unique records remained for title and abstract screening. Of these, 3,190 were excluded due to irrelevance, duplication, or failure to meet the inclusion criteria. Subsequently, 35 full-text articles were assessed for eligibility. Among them, 22 studies were excluded due to reasons such as overlapping patient cohorts, insufficient or unavailable outcome data, small sample sizes, or incomplete reporting. Ultimately, 13 studies met the inclusion criteria and were included in the meta-analysis.

### 3.2 Basic characteristics of the included literature

A total of 13 studies were included in this meta-analysis, comprising 5 RCTs and 8 single-arm studies, enrolling a total of 2,447 patients with mTNBC or HR+/HER2- mBC or early-stage breast cancer patients. These studies were conducted across various countries, including the United States, China, France, and Japan. All studies administered SG at a standard dose of 10 mg/kg intravenously on days 1 and 8 of a 21-day cycle. In the RCTs, SG was primarily compared to monotherapy such as eribulin, capecitabine, gemcitabine, or vinorelbine. One RCT additionally evaluated SG in combination with pembrolizumab versus SG monotherapy. Two single-arm trials investigated SG in combination with the PARP inhibitor talazoparib. Patients included in these trials were generally heavily pretreated. In the RCTs, most participants had received at least two prior systemic therapies, including taxanes and/or CDK4/6 inhibitors in the metastatic setting. Across studies, the median age of patients ranged from 48.5 to 57 years, while median follow-up durations varied between 6.1 and 18.9 months. Reported endpoints included PFS, OS, ORR, clinical benefit rate (CBR), duration of response (DOR), pathologic complete response (pCR) and safety outcomes, including AEs. Detailed baseline characteristics of the included studies are presented in **Table 1**.

**Table 1:** Baseline characteristics of included studies.

Country	First author	Year	Registration number	Study design	Phase	SG (N)	Control (N)	SG dose and schedule	Control therapy	Cancer stage	Molecular subtype	Prior lines of treatment	Median age of SG group (years)	Median age of control group (years)	Median follow-up time (months)	Outcomes
America	Aditya Bardia (28)	2024	NCT02574455	RCT	III	267	262	SG (10 mg/kg IV, 21-day cycle)	single-agent chemotherapy	IV	TNBC	≥2 prior standard chemotherapy (≥ 1 in the metastatic setting)	54 (27-82)	53 (27-81)	11.2 (0.3-30.8)	PFS, OS, ORR, AEs
America	Hope S Rugo (23)	2023	NCT03901339	RCT	III	272	271	SG (10 mg/kg IV, 21-day cycle)	single-agent chemotherapy	IV	HR+/HER2-	≥1 previous therapy and ≥2 chemotherapy for metastatic disease	57 (49-65)	55 (48-63)	12.5 (6.4-18.8)	PFS, OS, ORR, AEs
China	Binghe Xu	2024	NCT04639986	RCT	III	166	165	SG (10 mg/kg IV, 21-day cycle)	single-agent chemotherapy	IV	HR+/HER2-	≥2 prior systemic therapies (≥ 1 in the metastatic setting)	53 (32-72)	51 (28-79)	13.4 (0.1-28.7)	PFS, OS, ORR, AEs
America	Aditya Bardia (24)	2024	NCT02574455	RCT	III	235	233	SG (10 mg/kg IV, 21-day cycle	single-agent chemotherapy	IV	TNBC	≥2 previous standard chemotherapy regimens	54 (29-82)	53 (27-81)	17.7 (5.8-28.1)	PFS, OS, ORR, AEs
America	Ana Garrido-Castro	2024	NCT04448886	RCT	II	52	52	SG 10 mg/kg IV, plus pembrolizumab 200 mg IV, 21-day cycle	SG (10 mg/kg IV, 21-day cycle)	IV	HR+/HER2-	≥1 prior endocrine therapy and 0–1 chemotherapy for metastatic disease	57 (27-81)	NA	9.2 (NA)	PFS, OS, ORR, AEs
America	Aditya Bardia	2019	NCT01631552	Single-arm	I/II	108	NA	SG (10 mg/kg IV, 21-day cycle)	NA	IV	TNBC	≥2 previous anticancer regimens	55 (31-80)	NA	9.7 (0.3-36.5)	PFS, OS, ORR, AEs, CBR
America	K. Kalinsky	2020	NCT01631552	Single-arm	I/II	54	NA	SG (10 mg/kg IV, 21-day cycle)	NA	IV	HR+/HER2-	≥1 prior endocrine therapy and chemotherapy in the metastatic setting	54 (33-79)	NA	11.5(NA)	PFS, OS, ORR, AEs, DOR
America	L. M. Spring et.al	2023	NCT04230109	Single-arm	NA	50	NA	SG (10 mg/kg IV, 21-day cycle)	NA	≤III	TNBC	previously untreated	48.5 (31-77)	NA	18.9 (16.3-21.9)	EFS, ORR, AEs, pCR
America	Rachel Abelman	2024	NCT04039230	Single-arm	II	26	NA	SG 10mg/kg IV, plus talazoparib, 21-day cycle	NA	IV	TNBC	NA	54 (35-81)	NA	NA	PFS, OS, ORR, AEs, CBR
America	Aditya Bardia	2017	NCT01631552	Single-arm	I/II	69	NA	SG (10 mg/kg IV, 21-day cycle)	NA	IV	TNBC	≥1 prior standard line of therapy	56 (31-81)	NA	16.6 (NA)	PFS, OS, ORR, AEs
France	Alexandre Moura	2023	NA	Single-arm	NA	99	NA	SG (10 mg/kg IV, 21-day cycle)	NA	IV	TNBC	2 prior systemic treatments with ≥1 in the advanced setting	55 (26-89)	NA	9.7 (NA)	PFS, OS, ORR, AEs
Japan	Yoichi Naito	2024	NCT05101096	Single-arm	I/II	36	NA	SG (10 mg/kg IV, 21-day cycle)	NA	IV	TNBC	≥2 prior prior standard-of-care chemotherapy	50 (29–73)	NA	6.1 (NA)	PFS, ORR, AEs
America	Aditya Bardia (28)	2024	NCT04039230	Single-arm	Ib	30	NA	SG 10 mg/kg IV, plus talazoparib 1mg, 21-day cycle	NA	IV	TNBC	at least 2 weeks beyond prior treatment	NA	NA	NA	PFS, ORR, AEs

RCT, randomized controlled trial; TNBC, triple-negative breast cancer; HR+/HER2-, hormone receptor-positive and human epidermal growth factor receptor 2-negative; ORR, objective response rate; OS, overall survival; PFS, progression-free survival; DOR, duration of response; CBR, clinical benefit rate; AEs, adverse events; pCR, pathologic complete response. NA, not available.

### 3.3 Quality assessment

**Figure 2:**
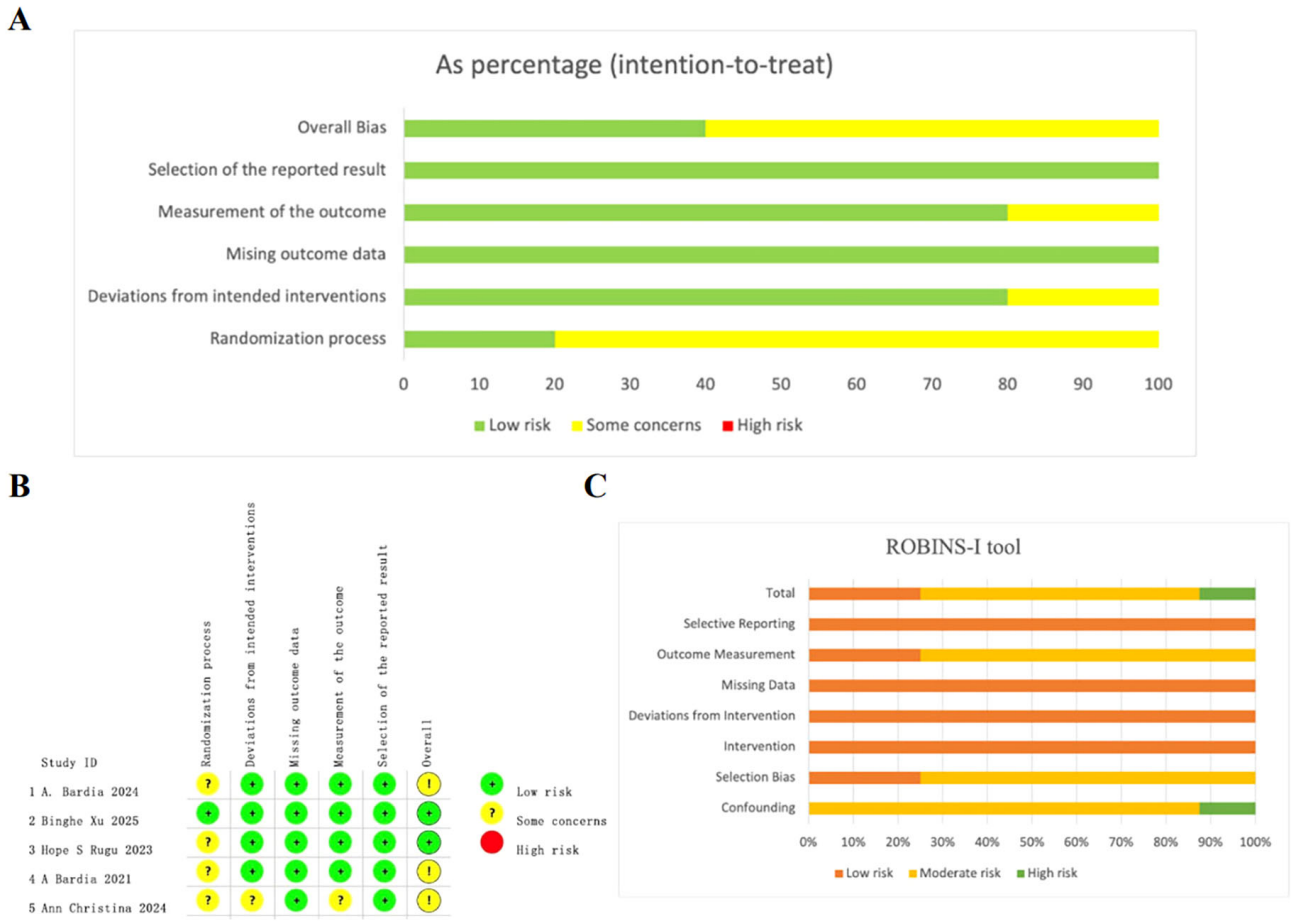
Quality assessment of enrolled studies. **(A)** Overall quality assessment of 5 RCTs using Revised cochrane risk of bias tool. **(B)** Detailed assessment of the risk of bias for RCTs. **(C)** Overall quality assessment of single-arm studies via the ROBINS-I tool.

Overall, the quality assessment of 5 RCTs and 8 single-arm studies was well reported. For the 5 RCTs, the overall risk of bias was assessed as low (**Figure 2A**). Among them, 4 RCTs were open-label RCTs and thus were rated as having “some concerns” in randomization process. A detailed assessment of the risk of bias for each study is presented in **Figure 2B**.

For the included single-arm studies, the risk of bias was assessed using the ROBINS-I tool. Among the 8 evaluated studies, two were rated as low risk, five as moderate risk, and one as high risk of bias. The most frequent source of bias was confounding, mainly due to insufficient adjustment for baseline characteristics or prognostic factors. Biases related to selection and outcome measurement were also common, particularly in studies lacking blinding or standardized outcome definitions. A summary of the ROBINS-I assessments for each study is provided in **Figure 2C** and **Supplementary Table 2**.

### 3.4 Efficacy

All 13 studies reported the efficacy outcomes of SG monotherapy in the treatment of breast cancer, primarily assessed using ORR, OS, and PFS. Given the methodological differences between RCTs and single-arm studies, we performed separate meta-analyses for each study type.

#### 3.4.1 Objective response rate

**Figure 3:**
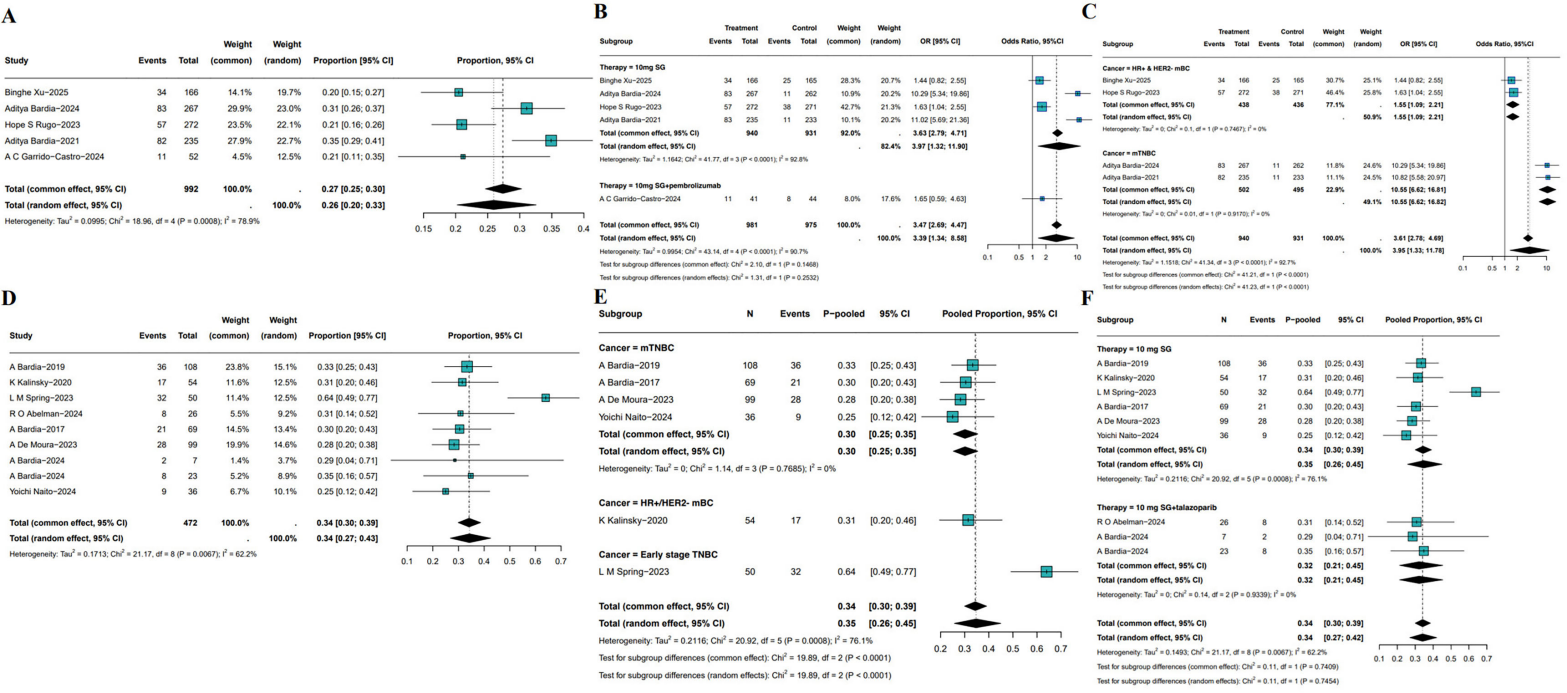
Pooled ORR of SG in breast cancer patients. **(A)** Pooled ORR of 5 RCTs. **(B, C)** Subgroup analysis based on therapy **(B)** and molecular subtypes **(C)**. **(D)** Pooled ORR of single-arm studies. **(E, F)** Subgroup analysis of single-arm studies based on molecular subtypes **(E)** and treatment **(F)**.

All 5 RCTs reported ORR as the primary indicator of clinical activity. In total, 1,779 patients (SG monotherapy: n=808; TPC: n=919, SG plus pembrolizumab: n=52) were enrolled. The pooled ORR across all RCTs was 26% (95%CI: 20%-33%; I²=78.9%; P = 0.0008), reflecting the consistent anti-tumor efficacy of SG in breast cancer (**Figure 3A**). Compared with TPC, SG monotherapy demonstrated a significantly higher ORR (OR = 3.97, 95%CI: 1.32-11.90, I²=92.8%, P<0.05). Furthermore, SG in combination with pembrolizumab showed a numerically higher ORR than SG monotherapy (**Figure 3B**), which suggests a potential synergistic effect. Due to the substantial heterogeneity among studies, sensitivity and subgroup analyses were performed. Sensitivity analysis demonstrated that the exclusion of any single study did not materially alter the pooled effect size and confidence intervals (**Supplementary Figure S1**). Subgroup analyses based on molecular subtypes revealed differential treatment benefits (**Figure 3C**). In patients with HR+/HER2- mBC, SG significantly improved ORR compared to TPC (OR = 1.55, 95%CI: 1.09-2.21, P = 0.75, I²=0%). Notably, SG conferred a substantially greater benefit in patients with mTNBC (OR: 10.55, 95%CI: 6.62-16.82, P = 0.92, I²=0%).

8 single-arm studies reported ORR, the pooled ORR of SG was 34% (95%CI: 27%-43%, **Figure 3D**). However, heterogeneity was substantial (I²=62.2%, P = 0.0067). Sensitivity analysis identified L. M. Spring’s study as a major contributor to heterogeneity. Exclusion of this study markedly reduced heterogeneity and resulted in a more symmetrical funnel plot (**Supplementary Figure S2**). Subgroup analysis based on disease stage and molecular subtypes demonstrated a significantly higher pooled ORR of 64% (95%CI: 49%-77%) in patients with early-stage TNBC, compared with 30% (95%CI: 25%-35%) in HR+/HER2- mBC and 31% (95%CI: 20%-46%) in mTNBC (**Figure 3E**). Further analysis stratified by treatment regimen showed no significant difference between SG monotherapy (pooled ORR: 35%, 95% CI: 26%-45%) and SG combined with talazoparib (pooled ORR: 32%, 95%CI: 21%-45%, **Figure 3F**). These findings underscore the need for additional clinical evidence to determine whether combining SG with immune checkpoint inhibitors or other agents confers incremental therapeutic benefit.

#### 3.4.2 Overall survival

**Figure 4:**
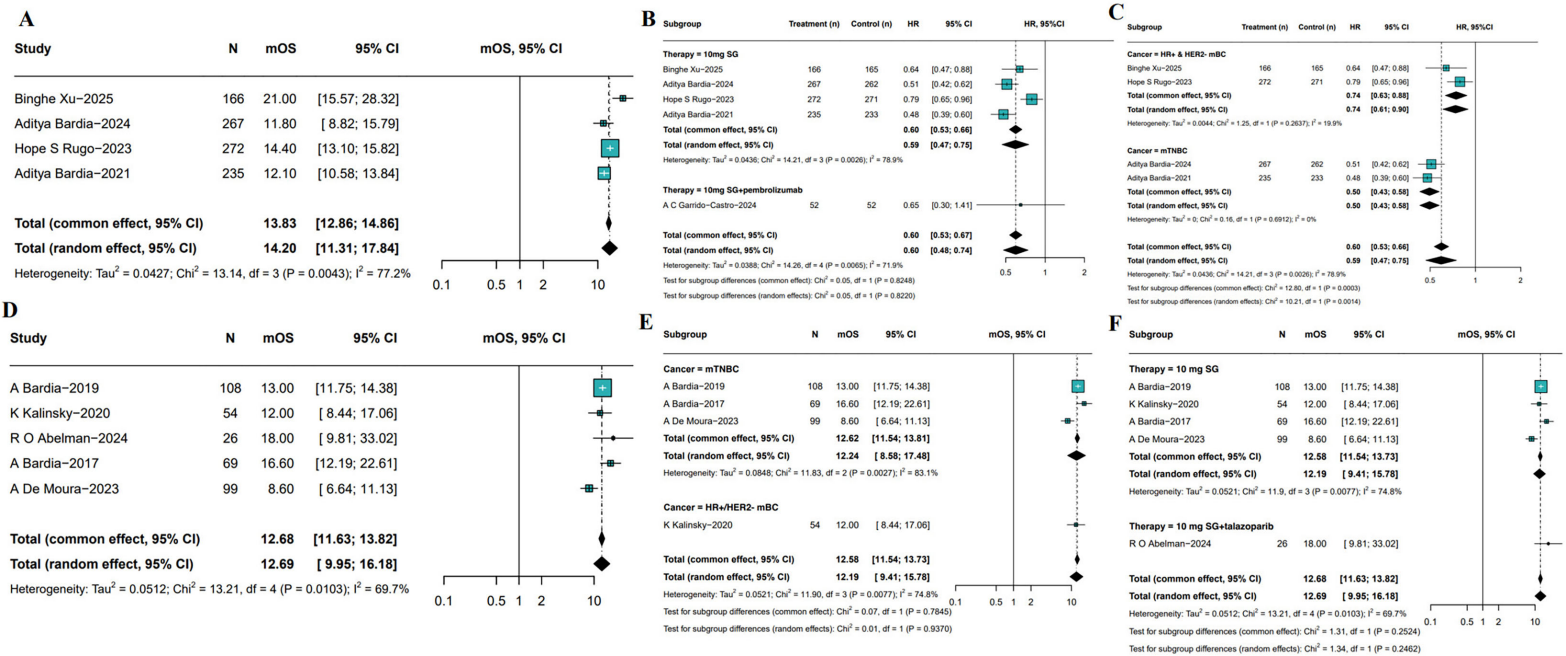
Pooled mOS of included studies. **(A)** The pooled mOS of RCTs. **(B, C)** Subgroup analyses based on therapy **(B)** and molecular subtypes **(C)**. **(D)** Pooled mOS from single-arm studies. **(E, F)** Subgroup analyses of single-arm studies based on molecular subtypes **(E)** and treatment regimens **(F)**.

5 RCTs have reported OS outcomes associated with SG monotherapy in patients with breast cancer. The pooled median OS (mOS) was 14.20 month (95%CI: 11.31-17.84, I²=77.2%, P = 0.0043; **Figure 4A**). As depicted in **Figure 4B**, SG significantly improved OS compared with treatment of physician’s choice (TPC) treatment (HR: 0.59, 95%CI: 0.47-0.75, I²=78.9%, P = 0.0026). Sensitivity analysis demonstrated consistent results, as exclusion of any single study did not materially affect the overall estimate or confidence intervals (**Supplementary Figure S3**). Besides, the combination of SG and pembrolizumab also demonstrated an OS advantage (HR: 0.65, 95%CI: 0.30-1.41). Subgroup analyses showed a consistent OS benefit across populations (**Figure 4C**). In HR+/HER2- mBC, SG treatment was associated with improved OS (HR: 0.74, 95% CI: 0.63–0.88, I²=19.9%, P = 0.2637). Similar findings were observed in patients with mTNBC (HR: 0.50, 95%CI: 0.43-0.58, I²=0%, P = 0.6912).

The meta-analysis of 5 single-arm studies demonstrated pooled mOS was 12.69 months (95%CI: 9.95-16.18, **Figure 4D**) among SG-treated breast cancer patients with moderate heterogeneity (I²=69.7%, P = 0.0103). Further subgroup analyses by tumor subtype and treatment regimen revealed mOS was 12.24 months (95%CI: 8.58-17.48) for mTNBC patients and 12.00 months (95%CI: 8.44-17.06) for HR+/HER2- mBC patients (**Figure 4E**). Additionally, patients receiving SG combined with immune checkpoint inhibitors (ICI) had a longer mOS of 18.00 months (95%CI: 9.81-33.02) compared to 12.19 months (95%CI: 9.41-15.78) in those treated with SG monotherapy (**Figure 4F**). Although heterogeneity remained, these results suggest a potential survival benefit with SG plus immunotherapy, which warrants further investigation in future studies. Sensitivity analyses confirmed the stability of the pooled mOS estimates (**Supplementary Figure S4**).

#### 3.4.3 Progression free survival

**Figure 5:**
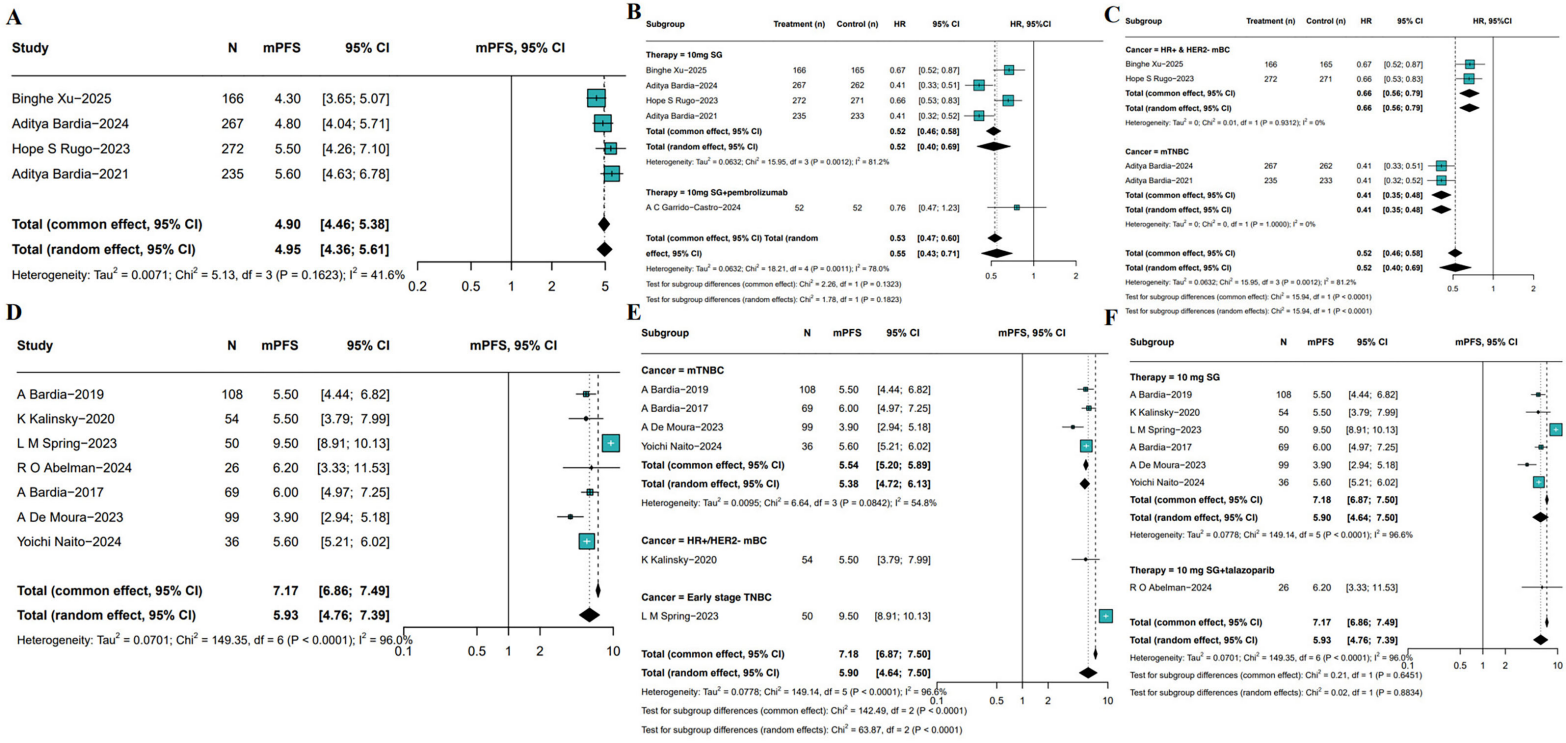
Pooled mPFS of SG in breast cancer patients. **(A)** Pooled mPFS from 5 RCTs. **(B, C)** Subgroup analyses based on therapy **(B)** and molecular subtypes **(C)**. **(D)** Pooled mPFS from single-arm studies. **(E, F)** Subgroup analyses of single-arm studies based on molecular subtypes **(E)** and treatment regimens **(F)**.

PFS outcomes associated with SG monotherapy were evaluated across both RCTs and single-arm studies. In RCTs, the pooled median PFS (mPFS) was 4.95 months (95%CIs: 4.36-5.61, I^2^ = 41.6%, P = 0.1623; **Figure 5A**). Subgroup analysis (**Figure 5B**) indicated SG significantly prolonged PFS compared to control group (HR: 0.52, 95%CI: 0.40-0.69, I^2^ = 81.2%, P = 0.0012), as well as SG and pembrolizumab group combined with SG monotherapy (HR: 0.76, 95%CI: 0.47-1.23). Sensitivity analyses using a leave-one-out approach confirmed the robustness of the results (**Supplementary Figure S5**). Subgroup analyses stratified by tumor subtype showed consistent PFS benefits (**Figure 5C**). In patients with HR+/HER2- mBC, SG was associated with improved PFS (HR: 0.66, 95%CI: 0.56-0.79; I²=0%, P = 0.9312). A greater benefit was observed in those with mTNBC (HR: 0.41, 95%CI: 0.35-0.48; I²=0%, P = 1.0000).

Meta-analysis of single-arm studies revealed a pooled mPFS of 5.93 months (95%CI: 4.76-7.39, I²=96.0%, P<0.0001) among SG-treated breast cancer patients (**Figure 5D**). To identify potential sources of heterogeneity, further subgroup analyses were conducted based on tumor subtype and treatment regimen (**Figure 5E**). Results showed mPFS was 5.50 months (95% CI: 3.79-7.99, I²=45.3%) in HR+/HER2- mBC patients, 5.38 months (95%CI: 4.72-6.13, I²=54.8%) in mTNBC patients, and a notably longer mPFS of 9.50 months (95%CI: 8.91-10.13) in early-stage breast cancer patients. Moreover, the mPFS of patients receiving SG combined with immunotherapy was 6.20 months (95%CI: 3.33-11.53, **Figure 5F**), which SG-monotherapy treated patients exhibited pooled mPFS was 5.90 months (95%CI: 4.64-7.50, I²=96.6%, P<0.001). Sensitivity analysis confirmed the stability of the pooled mPFS estimates (**Supplementary Figure S6**).

### 3.5 Adverse events (grade ≥3)

**Figure 6:**
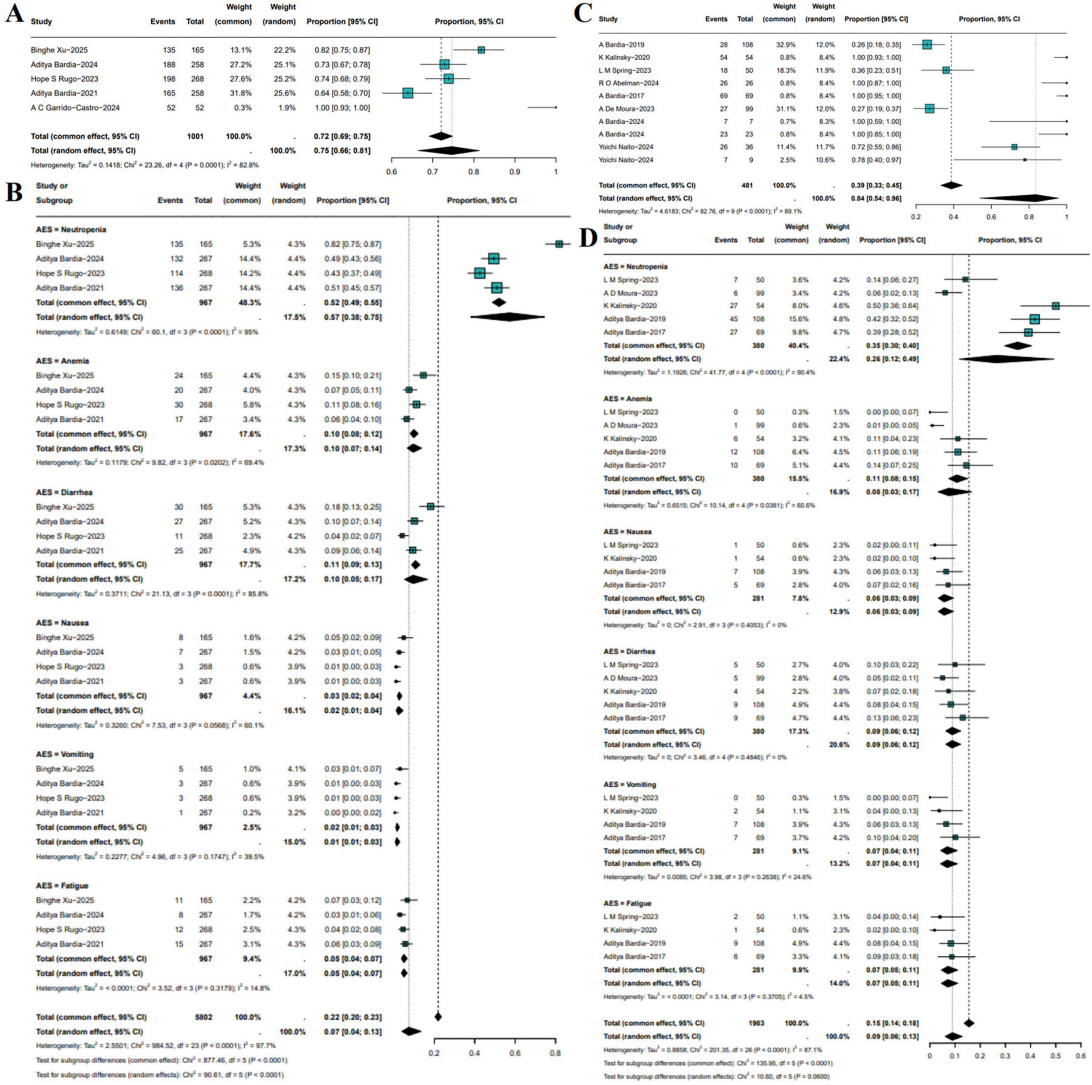
Incidence and subgroup analysis of treatment-related grade ≥3 adverse events (AEs) with SG. **(A)** Pooled incidence in RCTs. **(B)** Subgroup analysis of grade ≥3 AEs in RCTs. **(C)** Pooled incidence in single-arm studies. **(D)** Subgroup analysis of grade ≥3 AEs in single-arm studies.

Treatment-related grade ≥3 AEs were summarized across both RCTs and single-arm studies. In RCTs, meta-analysis showed that the pooled incidence of grade ≥3 AEs in patients receiving SG monotherapy was 75% (95%CI: 66%-81%, I²=82.8%, P = 0.0001; **Figure 6A**). Subgroup analysis demonstrated that SG was associated with a significantly increased risk of neutropenia (OR: 0.57, 95%CI: 0.38-0.75), whereas the incidences of other grade ≥3 AEs were relatively low (**Figure 6B**). In single-arm studies, the pooled incidence of grade ≥3 AEs was 84% (95%CI: 54%-96%, I²=89.1%, P<0.0001; **Figure 6C**). Subgroup analysis indicated that neutropenia remained the most frequent grade ≥3 AE, occurring in 26% of patients, followed by anemia, diarrhea, nausea, vomiting, and fatigue, each with relatively lower and comparable incidence rates (**Figure 6D**). Sensitivity analyses for both RCTs and single-arm studies yielded consistent results, which confirms the robustness of the findings (**Supplementary Figures S7**, **S8**).

### 3.6 Subgroup analyses

Given the high heterogeneity observed in the outcomes, subgroup analyses were conducted based on publication year, age, prior treatment, country, and follow-up duration. In RCTs, ORR (publication year: P<0.001; age: P<0.001; prior treatment: P<0.001), OS (publication year: P = 0.003; age: P = 0.001; prior treatment: P = 0.006), and PFS (publication year: P = 0.017; age: P<0.001; prior treatment: P = 0.001) differed significantly among subgroups defined by publication year, age, and prior treatment. No significant differences were observed across subgroups by country or follow-up duration (P>0.05), and AEs showed consistent results across all subgroups (P>0.05). The details were showed in **Table 2**.

**Table 2:** Subgroup analysis for RCTs.

Subgroups	Categories	ORR					OS					PFS					AES				
		N	OR	95% CI	I²	*P*	N	HR	95% CI	I²	*P*	N	HR	95% CI	I²	*P*	*N*	OR	95% CI	I²	*P*
Publication year	2025	1	1.44	0.82-2.25	/	<0.001	1	0.64	0.47-0.88	/	0.003	1	0.67	0.52-0.87	/	0.017	1	1.97	1.18-3.31	/	0.631
	2024	2	4.31	0.72-25.87	88		2	0.52	0.43-0.63	0		2	0.54	0.29-0.98	81		1	1.46	0.99-2.16	/	
	2023	1	1.63	1.04-2.55	/		1	0.79	0.65-0.95	/		1	0.66	0.53-0.83	/		1	1.87	1.29-2.71	/	
	2021	1	11.02	5.69-21.63	/		1	0.48	0.39-0.60	/		1	0.41	0.32-0.52	/		1	2.05	1.42-2.95	/	
Age	≤55	3	2.91	0.81-10.42	92	<0.001	3	0.62	0.46-0.84	82	0.001	3	0.57	0.41-0.78	80	<0.001	3	1.96	1.55-2.47	0	0.205
	>55	2	4.31	0.72-25.87	88		2	0.52	0.43-0.63	0		2	0.54	0.29-0.98	81		1	1.46	0.99-2.16	/	
Prior treatment	CDK4/6 inhibitor	2	1.55	1.09-2.21	0	<0.001	2	0.74	0.63-0.88	20	0.006	2	0.66	0.56-0.79	0	0.001	2	1.9	1.42-2.57	0	0.679
	CT	2	10.65	6.68-16.97	0		2	0.50	0.43-0.58	0		2	0.41	0.35-0.48	0		2	1.75	1.34-2.28	34	
	CT+ET	1	1.65	0.59-4.63	/		1	0.65	0.30-1.41	/		1	0.76	0.47-1.23	/		/	/	/	/	
Country	America	4	4.24	1.47-12.29	91	0.079	4	0.59	0.45-0.77	79	0.822	4	0.52	0.39-0.70	79	0.182	3	1.79	1.44-2.22	0	0.859
	China	1	1.44	0.82-2.25	/		1	0.64	0.47-0.88	/		1	0.67	0.52-0.87	/		1	1.97	1.18-3.31	/	
Follow-up duration	≤12 months	2	1.49	0.90-2.45	0	0.831	2	0.64	0.48-0.86	0	0.465	2	0.69	0.55-0.86	0	0.772	1	1.97	1.18-3.31	/	0.729
	>12 months	3	5.57	1.60-19.43	94		3	0.58	0.43-0.79	86		3	0.48	0.35-0.66	82		2	1.79	1.44-2.22	0	

CTs, Chemotherapy; ET, Endocrine therapy.

In single-arm trials, subgroup analyses revealed that prior treatment lines significantly influenced pooled ORR (≤1 line: 0.32; ≥2 lines: 0.28; untreated: 0.33) and mPFS (≤1 line: 5.74; ≥2 lines: 5.03; untreated: 9.50), whereas mOS differed significantly across publication years (P = 0.010). No significant differences were observed for age and follow-up duration (P>0.05). Adverse events were generally consistent across subgroups, although some variation was noted by country (P<0.001). Detailed results are presented in **Table 3**, which highlights potential sources of heterogeneity.

**Table 3:** Subgroup analysis for single-arm trials.

Subgroups	Categories	ORR					OS					PFS					AES				
		N	Pooled	95% CI	I²	*P*	N	mOS	95% CI	I²	*P*	N	mPFS	95% CI	I²	*P*	N	Pooled	95% CI	I²	*P*
Publication year	2024	4	0.29	0.21-0.39	0	0.824	1	8.60	6.64-11.13	/	0.010	2	5.61	5.22-6.02	0	0.969	5	0.88	0.80-0.93	0	0.074
	2023	2	0.45	0.22-0.70	94		1	16.60	12.19-22.61	/		2	6.16	2.57-14.72	97		2	0.30	0.23-0.38	16	
	2020	1	0.31	0.20-0.46	/		1	18.00	9.81-33.02	/		1	5.50	3.79-7.99	/		1	1.00	0.93-1.00	/	
	2019	1	0.33	0.25-0.43	/		1	12.00	8.44-17.06	/		1	5.50	4.44-6.82	/		1	0.26	0.18-0.35	/	
	2017	1	0.30	0.20-0.43	/		1	13.00	11.75-14.38	/		1	6.00	4.97-7.25	/		1	1.00	0.95-1.00	/	
Age	≤55	8	0.35	0.27-0.44	66	0.555	4	11.82	9.19-15.21	70	0.095	5	5.90	4.54-7.66	97	0.917	9	0.92	0.49-0.99	73	0.999
	>55	1	0.30	0.20-0.43	/		1	16.60	12.19-22.61			1	6.00	4.97-7.25	/		1	1.00	0.95-1.00	/	
Prior treatment	≤1line ST	3	0.32	0.26-0.38	0	0.001	3	13.21	12.05-14.49	19	0.752	3	5.74	5.03-6.56	0	<0.001	3	0.65	0.59-0.71	0	0.210
	≥2 lines ST	3	0.28	0.22-0.35	0		2	11.80	5.76-24.15	79		3	5.03	3.83-6.59	67		4	0.80	0.32-0.97	88	
	untreated	1	0.33	0.19-0.52	/		/	/	/	/		1	9.50	8.91-10.13	/		1	0.36	0.23-0.51	/	
Country	America	7	0.37	0.28-0.46	66	0.294	4	13.31	12.15-14.58	13	0.002	5	6.58	5.12-8.44	91	0.021	7	0.67	0.62-0.72	0	<0.001
	France	1	0.28	0.20-0.38	/		1	8.60	6.64-11.13	/		1	3.90	2.94-5.18	/		1	0.27	0.19-0.37	/	
	Japan	1	0.25	0.12-0.42	/		/	/	/	/		1	5.60	5.21-6.02	/		2	0.73	0.59-0.84	0	
Follow-up duration	≤12 months	8	0.35	0.27-0.44	66	0.404	3	11.17	8.61-14.50	77	0.100	4	6.71	4.90-9.21	90	0.204	4	0.74	0.15-0.98	87	0.402
	>12 months	1	0.30	0.20-0.43	/		1	16.60	12.19-22.61	/		3	5.23	4.18-6.53	70		2	0.73	0.64-0.80	0	

ST, systemic therapy.

### 3.7 Publication bias

**Figure 7:**
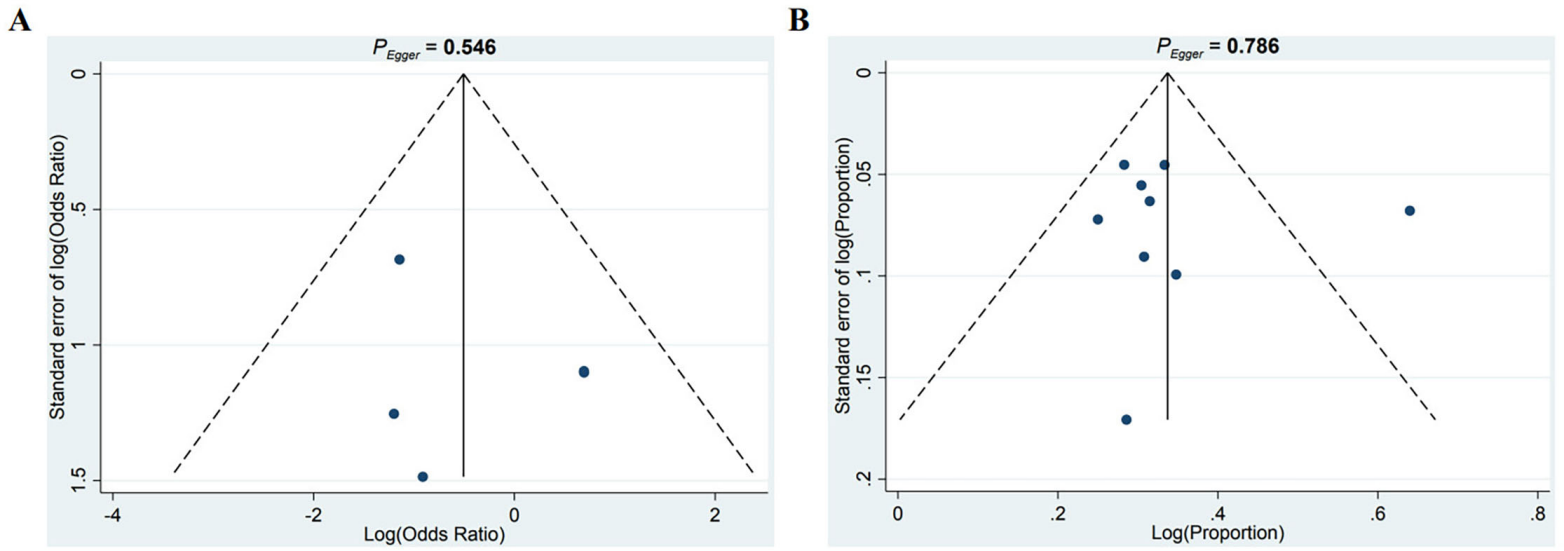
Publication bias assessment. **(A, B)** Funnel plots of RCTs **(A)** and single-arm studies **(B)**.

To evaluate potential publication bias, funnel plots were generated by plotting effect sizes against the standard errors of log HRs. The resulting plot showed approximate symmetry, suggesting no substantial publication bias. Additionally, Egger’s test (P = 0.546, P = 0.786) did not indicate significant bias (**Figures 7A, B**).

## 4 Discussion

SG is a novel ADC targeting the transmembrane glycoprotein TROP-2, which is overexpressed in various solid tumors and has emerged as a promising target for next-generation ADC therapies (21). By conjugating a humanized anti-TROP-2 antibody with the topoisomerase I inhibitor SN-38, SG delivers potent cytotoxicity directly to TROP-2-expressing tumor cells, thereby combining targeted specificity with robust anti-tumor activity (22). SG has garnered significant clinical attention, particularly in mTNBC where treatment options are limited and outcomes remain poor (23). This meta-analysis systematically included 13 clinical trials (5 RCTs and 8 single-arm studies) to provide the most comprehensive synthesis to date of SG’s efficacy and safety across different molecular subtypes, treatment stages, and combination regimens in breast cancer. Through detailed subgroup analyses by study design, molecular subtype, and treatment context, this meta-analysis not only validates the efficacy of SG in advanced breast cancer but also highlights critical factors to refine patient stratification, optimize therapeutic sequencing, and inform the development of future clinical trials.

The pooled ORR from RCTs (26%, 95%CIs: 20%-33%) and single-arm trials (34%, 95%CIs: 27%-43%) consolidates the view that SG delivers clinically meaningful tumor shrinkage in both TNBC and HR+/HER2- mBC. The significantly higher ORR in mTNBC compared to HR+/HER2- mBC (OR: 10.55(95%CIs:6.62-16.82) *vs*. 1.55(95%CIs: 1.09-2.21) in RCTs) is biologically plausible. TNBC tend to express higher levels of Trop-2 (24, 25) which was the antigen targeted by SG, and may be more sensitive to the DNA-damaging payload SN-38 due to intrinsic defects in DNA repair (26). In contrast, HR+/HER2- tumors often harbor endocrine resistance mechanisms and reduced proliferation rates, potentially attenuating ADC efficacy (27). Our findings are consistent with pivotal trials such as ASCENT (28) and TROPiCS-02 (29). Moreover, our meta-analysis extends these observations by integrating real-world and early-stage data. For early-stage TNBC, single-arm neoadjuvant studies reported an ORR of 64% and a pCR rate of 30% (30). Although these results suggest SG can be active in this setting, the small sample size and single-arm design mean the findings are preliminary and should be interpreted with caution. Further studies are needed to clarify its role in neoadjuvant or adjuvant therapy. In HR+/HER2- mBC, SG still demonstrated clinically relevant benefit (ORR = 1.55(95%CIs: 1.09-2.21); OS HR = 0.74, 95%CIs: 0.61-0.90), underscoring TROP-2 as a potential cross-subtype therapeutic target, although biomarker-driven stratification remains necessary to address inter-patient heterogeneity (31).

SG’s survival benefit is not merely a function of tumor shrinkage. In RCTs, the mOS of SG (HR: 0.59, 95CIs: 0.47-0.75) versus TPC mirrors ASCENT’s results (28) and reinforces SG’s ability to extend survival even in late-line patients. Importantly, our analysis shows that this OS advantage is preserved across molecular subtypes, though the absolute gain is greater in TNBC. Median PFS in RCTs was modestly prolonged (HR: 0.52, 95%CIs: 0.40-0.69), with a striking difference between TNBC (HR: 0.41, 95%CIs: 0.35-0.48) and HR+/HER2- mBC (HR: 0.66, 95%CIs: 0.56-0.79). These disparities underscore the biological heterogeneity of breast cancer and suggest that Trop-2 expression (32) and tumor microenvironment (33) may jointly determine treatment sensitivity. Combination strategies with ICIs or PARP inhibitors remain exploratory and are based on small patient cohorts. Meta-subgroup analyses showed that adding these agents to SG did not significantly increase ORR, though median OS appeared numerically higher in the SG+ICI subgroup (18 months *vs*. 12.2 months for SG monotherapy) (34). This is mechanistically plausible, as the SN-38 payload can trigger immunogenic cell death, enhancing tumor immunogenicity, promoting T cell infiltration, and increasing ICI responsiveness (35). However, these observations are preliminary and should be interpreted cautiously. Early studies indicate a potential immunologic interaction of SG, but the small sample sizes prevent firm conclusions. Ongoing early-phase trials including ASCENT-04 (36) and OptimICE-RD (37) are designed to explore the efficacy of SG in combination with immunotherapy in both metastatic and residual TNBC. Nevertheless, combination regimens currently face challenges including high efficacy heterogeneity, difficulty in identifying responsive patient populations and absence of robust predictive biomarkers (38, 39). Future research should integrate immune microenvironment profiling and immune-sensitivity markers to optimize combination strategies.

Our pooled safety analysis confirms that grade ≥3 AEs are common with SG, with neutropenia being the most frequent (26% in single-arm studies and 57% in RCTs), followed by diarrhea, fatigue, and nausea, predominantly related to the SN-38-mediated irinotecan-like toxicity (40). Although the overall incidence of toxicity is relatively high, severe non-hematologic events remain infrequent, and most AEs are manageable with dose modifications, granulocyte colony-stimulating factor (G-CSF) prophylaxis, and supportive care (41, 42). Dose modification strategies for SG generally involve stepwise dose reductions from the standard 10 mg/kg on days 1 and 8 of a 21-day cycle to 7.5 mg/kg or 5 mg/kg, as well as temporary treatment interruptions for grade ≥3 toxicities until recovery, with resumption at the same or reduced dose depending on severity and recurrence (43). These strategies combined with prophylactic interventions, which allows patients to maintain dose intensity while minimizing the risk of severe toxicity. Recent prospective data from the PRIMED trial (44) demonstrated that primary prophylactic administration of G-CSF and loperamide significantly reduced the incidence and severity of SG-related neutropenia and numerically lowered SG-related diarrhea, thereby decreasing rates of dose reductions (14%) and temporary treatment interruptions (30%), with no treatment discontinuations during the first two cycles. This contrasts favorably with the ASCENT and TROPiCS-02 trials, where neutropenia (any grade: 60-70%; grade ≥3: ~50%) and diarrhea (any grade: ~60%; grade ≥2: ~30%) were common, leading to dose reductions in 20-30%, interruptions in ~60%, and discontinuations in ~5% of patients (45, 46). For gastrointestinal toxicity, early intervention with loperamide, hydration, and electrolyte replacement remains the cornerstone of management (22). However, PRIMED (44) also reported that prophylactic loperamide was associated with increased constipation (46% *vs*. 17-37% in ASCENT and TROPiCS-02), underscoring the need to optimize dosing and scheduling of prophylactic regimens. In addition, current supportive care guidelines recommend antiemetic prophylaxis such as 5-HT3 receptor antagonists and/or NK1 receptor antagonists for SG-associated nausea and vomiting (47). Treatment discontinuations due to intolerance are uncommon, but in early-stage and combination regimens, cumulative toxicity should be monitored closely, particularly in patients with limited marrow reserve from prior therapies (48). Recent exposure-response analyses from IMMU-132–01 and ASCENT trials demonstrated that higher SG serum concentrations are associated with increased risk of AEs, particularly neutropenia, which is the only grade ≥3 toxicity significantly predicted by drug exposure (49). Emerging evidence suggests that the toxicity profile of SG may be linked to TROP-2 expression levels (50), hepatic function (51), and baseline bone marrow reserve (52), highlighting the potential for clinical risk stratification based on physiological and genetic factors. Importantly, SG does not demonstrate cumulative toxicity in heavily pretreated patients, thus supporting the tolerability even after multiple prior therapies. These findings highlight the importance of proactive AE prevention and early integration of supportive care protocols to maintain dose intensity, which has been associated with efficacy in prior ADC trials (44).

Our meta-analysis demonstrates that SG provides consistent clinical benefit in terms of ORR, OS, and PFS, with a toxicity profile that is generally manageable. Nevertheless, substantial heterogeneity was observed across studies. Publication year emerged as a consistent source of variability, with more recent studies (publication year: 2023-2025) generally reporting higher ORRs and longer OS compared with earlier trials, which may be attributed to improved patient selection, optimized sequencing of systemic therapies, and enhanced supportive care measures over time (53, 54). Patients with ≤1 prior line of systemic therapy consistently achieved better responses and longer survival than those heavily pretreated, suggesting that tumor sensitivity and preserved bone marrow reserve are critical determinants of ADC efficacy (55, 56). Patients’ age also contributed to heterogeneity in RCTs. This may be due to younger patients tend to better marrow reserve and organ function to tolerate full-dose therapy, while OS may not always be superior due to more aggressive tumor biology (57). Notably, age did not show a significant impact on efficacy outcomes in single-arm studies, which may reflect the inherent limitations of non-comparative designs, including selection bias and smaller sample sizes. Clinically, these findings underscore the importance of individualized treatment planning. Patient selection should consider prior therapy burden, age, and comorbidities to optimize ADC sequencing and minimize toxicity risk (58). Early monitoring of disease response and proactive management of AEs are essential, particularly in heavily pretreated or older patients. Additionally, heterogeneity across regions highlights the need for context-specific supportive care strategies and adherence to guideline-based monitoring to ensure patients achieve optimal outcomes in real-world practice (59).

Notably, while TROP-2 is the direct target of SG, clinical evidence suggests that its expression level is not a reliable standalone predictor of therapeutic efficacy (60). *Post-hoc* biomarker analyses from the phase III TROPiCS-02 trial demonstrated that SG significantly improved PFS and OS irrespective of Trop-2 gene mRNA expression levels (61, 62). Also, a pilot study by Kalinsky et al. (63) found no significant difference in Trop-2 H-score or staining percentage between ‘excellent responders’ and ‘non-responders’ to SG, though a lower percentage staining was associated with shorter PFS in that cohort. These findings suggest that Trop-2 expression may not be a strong standalone predictive biomarker, and SG’s antitumor efficacy likely depends on additional factors. Current investigations are evaluating composite predictive models incorporating TROP-2 immunohistochemistry (IHC) scoring (64), ADC internalization efficiency (65), CD8+ T cell infiltration levels (66), and dynamic changes in circulating tumor DNA (ctDNA) (67) to better predict treatment sensitivity. We also highlight that the predictive value of TROP-2 remains a limitation, as these models are not yet validated for routine clinical decision-making. Compared with other ADCs such as trastuzumab emtansine (T-DM1) and trastuzumab deruxtecan (T-DXd) which both target HER2-positive breast tumors (68), SG’s targeting of TROP-2-expressed across multiple breast cancer subtypes including TNBC and HR+ breast cancer, broadens the therapeutic applicability. Moreover, SG delivers the potent topoisomerase I inhibitor SN-38 which overcomes heterogeneous antigen expression and resistance mechanisms seen with other ADCs (55, 69). However, SG is associated with higher rates of hematologic toxicities such as neutropenia, requiring careful monitoring and supportive care, whereas agents like T-DXd may carry increased risks of interstitial lung disease (70). Clinicians selecting among ADC options must weigh these efficacy and safety profiles alongside tumor subtype, prior treatments, and patient comorbidities. Thus, SG represents a valuable option particularly in heavily pretreated mTNBC and HR+ breast cancer patients, where alternative targeted therapies are limited.

This meta-analysis has several limitations. Although the included RCTs had adequate sample sizes, most single-arm studies were based on relatively small cohorts, which potentially reduces the precision of pooled estimates and limits the generalizability of the findings to broader clinical practice. In addition, the inclusion of abstract-only data further restricted the interpretability of results and weakened external validity, as key clinical characteristics such as age distribution, prior therapy, and biomarker status were often unavailable. Heterogeneity in treatment regimens, particularly between SG monotherapy and combination approaches further complicates interpretation. Moreover, the absence of standardized endpoint definitions may have introduced variability in outcome assessment. Future research should prioritize large-scale, high-quality trials with standardized reporting and comprehensive stratified analyses to improve the robustness and generalizability of the evidence base.

## 5 Conclusion

This meta-analysis demonstrates that SG confers meaningful clinical benefit in breast cancer with significant improvements in ORR, OS, and PFS compared to single-agent chemotherapy, especially in mTNBC. The efficacy of SG was maintained across treatment settings and combination strategies, with a potential survival advantage when combined with immunotherapy. Although hematologic and gastrointestinal toxicities were common, the toxicity of SG were generally manageable. These findings support SG as an important therapeutic option for breast cancer, while highlighting the need for vigilant toxicity monitoring and further research to optimize patient selection and combination approaches.

## Data Availability

The original contributions presented in the study are included in the article/**Supplementary Material**. Further inquiries can be directed to the corresponding authors.

## Supplementary material

Supplementary Figure 1 Sensitivity analysis of ORR in RCTs by omitting one study at a time.

Supplementary Figure 2 Sensitivity analysis of ORR in single-arm studies.

Supplementary Figure 3 Sensitivity analysis of OS in RCTs.

Supplementary Figure 4 Sensitivity analysis of OS in single-arm studies.

Supplementary Figure 5 Sensitivity analysis of PFS in RCTs.

Supplementary Figure 6 Sensitivity analysis of PFS in single-arm studies.

Supplementary Figure 7 Sensitivity analysis of grade ≥3 AEs incidence in RCTs.

Supplementary Figure 8 Sensitivity analysis of grade ≥3 AEs incidence in single-arm studies.

## References

[1] Bray F Laversanne M Sung H Ferlay J Siegel RL Soerjomataram I . Global cancer statistics 2022: GLOBOCAN estimates of incidence and mortality worldwide for 36 cancers in 185 countries. CA Cancer J Clin. (2024) 74:229–63. doi: 10.3322/caac.21834, PMID: 38572751

[2] Anandasabapathy S Asirwa C Grover S Mungo C . Cancer burden in low-income and middle-income countries. Nat Rev Cancer. (2024) 24:167–70. doi: 10.1038/s41568-023-00659-2, PMID: 38332323

[3] Xiong X Zheng LW Ding Y Chen YF Cai YW Wang LP . Breast cancer: pathogenesis and treatments. Signal Transduct Target Ther. (2025) 10:49. doi: 10.1038/s41392-024-02108-4, PMID: 39966355 PMC11836418

[4] Leon-Ferre RA Goetz MP . Advances in systemic therapies for triple negative breast cancer. Bmj. (2023) 381:e071674. doi: 10.1136/bmj-2022-071674, PMID: 37253507

[5] Halim PA Hassan RA Mohamed KO Hassanin SO Khalil MG Abdou AM . Synthesis and biological evaluation of halogenated phenoxychalcones and their corresponding pyrazolines as cytotoxic agents in human breast cancer. J Enzyme Inhib Med Chem. (2022) 37:189–201. doi: 10.1080/14756366.2021.1998023, PMID: 34894967 PMC8667918

[6] Bai X Ni J Beretov J Graham P Li Y . Triple-negative breast cancer therapeutic resistance: Where is the Achilles' heel? Cancer Lett. (2021) 497:100–11. doi: 10.1016/j.canlet.2020.10.016, PMID: 33069769

[7] Behl A Wani ZA Das NN Parmar VS Len C Malhotra S . Monoclonal antibodies in breast cancer: A critical appraisal. Crit Rev Oncol Hematol. (2023) 183:103915. doi: 10.1016/j.critrevonc.2023.103915, PMID: 36702424

[8] Saini S Gulati N Awasthi R Arora V Singh SK Kumar S . Monoclonal antibodies and antibody-drug conjugates as emerging therapeutics for breast cancer treatment. Curr Drug Deliv. (2024) 21:993–1009. doi: 10.2174/1567201820666230731094258, PMID: 37519200

[9] Fu Z Li S Han S Shi C Zhang Y . Antibody drug conjugate: the "biological missile" for targeted cancer therapy. Signal Transduct Target Ther. (2022) 7:93. doi: 10.1038/s41392-022-00947-7, PMID: 35318309 PMC8941077

[10] Liu X Deng J Yuan Y Chen W Sun W Wang Y . Advances in Trop2-targeted therapy: Novel agents and opportunities beyond breast cancer. Pharmacol Ther. (2022) 239:108296. doi: 10.1016/j.pharmthera.2022.108296, PMID: 36208791

[11] Bessede A Peyraud F Besse B Cousin S Cabart M Chomy F . TROP2 is associated with primary resistance to immune checkpoint inhibition in patients with advanced non-small cell lung cancer. Clin Cancer Res. (2024) 30:779–85. doi: 10.1158/1078-0432.Ccr-23-2566, PMID: 38048058 PMC10870116

[12] Rosellini M Santoni M Mollica V Rizzo A Cimadamore A Scarpelli M . Treating prostate cancer by antibody-drug conjugates. Int J Mol Sci. (2021) 22:1551. doi: 10.3390/ijms22041551, PMID: 33557050 PMC7913806

[13] Hu Y Zhu Y Qi D Tang C Zhang W . Trop2-targeted therapy in breast cancer. biomark Res. (2024) 12:82. doi: 10.1186/s40364-024-00633-6, PMID: 39135109 PMC11321197

[14] Liu X Ma L Li J Sun L Yang Y Liu T . Trop2-targeted therapies in solid tumors: advances and future directions. Theranostics. (2024) 14:3674–92. doi: 10.7150/thno.98178, PMID: 38948057 PMC11209721

[15] Syed YY . Sacituzumab govitecan: first approval. Drugs. (2020) 80:1019–25. doi: 10.1007/s40265-020-01337-5, PMID: 32529410 PMC7288263

[16] Wahby S Fashoyin-Aje L Osgood CL Cheng J Fiero MH Zhang L . FDA approval summary: accelerated approval of sacituzumab govitecan-hziy for third-line treatment of metastatic triple-negative breast cancer. Clin Cancer Res. (2021) 27:1850–54. doi: 10.1158/1078-0432.Ccr-20-3119, PMID: 33168656

[17] Cumpston M Li T Page MJ Chandler J Welch VA Higgins JP . Updated guidance for trusted systematic reviews: a new edition of the Cochrane Handbook for Systematic Reviews of Interventions. Cochrane Database Syst Rev. (2019) 10:Ed000142. doi: 10.1002/14651858.Ed000142, PMID: 31643080 PMC10284251

[18] Page MJ McKenzie JE Bossuyt PM Boutron I Hoffmann TC Mulrow CD . The PRISMA 2020 statement: an updated guideline for reporting systematic reviews. Bmj. (2021) 372:n71. doi: 10.1136/bmj.n71, PMID: 33782057 PMC8005924

[19] Crocker TF Lam N Jordão M Brundle C Prescott M Forster A . Risk-of-bias assessment using Cochrane's revised tool for randomized trials (RoB 2) was useful but challenging and resource-intensive: observations from a systematic review. J Clin Epidemiol. (2023) 161:39–45. doi: 10.1016/j.jclinepi.2023.06.015, PMID: 37364620

[20] Igelström E Campbell M Craig P Katikireddi SV . Cochrane's risk of bias tool for non-randomized studies (ROBINS-I) is frequently misapplied: A methodological systematic review. J Clin Epidemiol. (2021) 140:22–32. doi: 10.1016/j.jclinepi.2021.08.022, PMID: 34437948 PMC8809341

[21] Rossi V Turati A Rosato A Carpanese D . Sacituzumab govitecan in triple-negative breast cancer: from bench to bedside, and back. Front Immunol. (2024) 15:1447280. doi: 10.3389/fimmu.2024.1447280, PMID: 39211043 PMC11357913

[22] Schlam I Tarantino P Tolaney SM . Managing adverse events of sacituzumab govitecan. Expert Opin Biol Ther. (2023) 23:1103–11. doi: 10.1080/14712598.2023.2267975, PMID: 37800595

[23] Adrada BE Moseley TW Kapoor MM Scoggins ME Patel MM Perez F . Triple-negative breast cancer: histopathologic features, genomics, and treatment. Radiographics. (2023) 43:e230034. doi: 10.1148/rg.230034, PMID: 37792593 PMC10560981

[24] Jeon Y Jo U Hong J Gong G Lee HJ . Trophoblast cell-surface antigen 2 (TROP2) expression in triple-negative breast cancer. BMC Cancer. (2022) 22:1014. doi: 10.1186/s12885-022-10076-7, PMID: 36153494 PMC9509625

[25] Sakach E Sacks R Kalinsky K . Trop-2 as a therapeutic target in breast cancer. Cancers (Basel). (2022) 14:5936. doi: 10.3390/cancers14235936, PMID: 36497418 PMC9735829

[26] Lee KJ Mann E Wright G Piett CG Nagel ZD Gassman NR . Exploiting DNA repair defects in triple negative breast cancer to improve cell killing. Ther Adv Med Oncol. (2020) 12:1758835920958354. doi: 10.1177/1758835920958354, PMID: 32994807 PMC7502856

[27] Samantasinghar A Sunildutt NP Ahmed F Soomro AM Salih ARC Parihar P . A comprehensive review of key factors affecting the efficacy of antibody drug conjugate. BioMed Pharmacother. (2023) 161:114408. doi: 10.1016/j.biopha.2023.114408, PMID: 36841027

[28] Bardia A Rugo HS Tolaney SM Loirat D Punie K Oliveira M . Final results from the randomized phase III ASCENT clinical trial in metastatic triple-negative breast cancer and association of outcomes by human epidermal growth factor receptor 2 and trophoblast cell surface antigen 2 expression. J Clin Oncol. (2024) 42:1738–44. doi: 10.1200/jco.23.01409, PMID: 38422473 PMC11107894

[29] Rugo HS Bardia A Marmé F Cortés J Schmid P Loirat D . Overall survival with sacituzumab govitecan in hormone receptor-positive and human epidermal growth factor receptor 2-negative metastatic breast cancer (TROPiCS-02): a randomised, open-label, multicentre, phase 3 trial. Lancet. (2023) 402:1423–33. doi: 10.1016/s0140-6736(23)01245-x, PMID: 37633306

[30] Kim SI Cassella CR Byrne KT . Tumor burden and immunotherapy: impact on immune infiltration and therapeutic outcomes. Front Immunol. (2020) 11:629722. doi: 10.3389/fimmu.2020.629722, PMID: 33597954 PMC7882695

[31] Bardia A Tolaney SM Punie K Loirat D Oliveira M Kalinsky K . Biomarker analyses in the phase III ASCENT study of sacituzumab govitecan versus chemotherapy in patients with metastatic triple-negative breast cancer. Ann Oncol. (2021) 32:1148–56. doi: 10.1016/j.annonc.2021.06.002, PMID: 34116144

[32] Weiten R Niemann M Below E Friker LL Ralser DJ Toma M . Preclinical evidence for the use of anti-Trop-2 antibody-drug conjugate Sacituzumab govitecan in cerebral metastasized castration-resistant prostate cancer. Cancer Med. (2024) 13:e7320. doi: 10.1002/cam4.7320, PMID: 38895886 PMC11185941

[33] Yan WL Lang TQ Yuan WH Yin Q Li YP . Nanosized drug delivery systems modulate the immunosuppressive microenvironment to improve cancer immunotherapy. Acta Pharmacol Sin. (2022) 43:3045–54. doi: 10.1038/s41401-022-00976-6, PMID: 36050519 PMC9712392

[34] Goldenberg DM Sharkey RM . Sacituzumab govitecan, a novel, third-generation, antibody-drug conjugate (ADC) for cancer therapy. Expert Opin Biol Ther. (2020) 20:871–85. doi: 10.1080/14712598.2020.1757067, PMID: 32301634

[35] Nucera S Conti C Martorana F Wilson B Genta S . Antibody-drug conjugates to promote immune surveillance: lessons learned from breast cancer. Biomedicines. (2024) 12:1491. doi: 10.3390/biomedicines12071491, PMID: 39062065 PMC11274676

[36] Bian L Zhang S Li M Yang J Yin Y . Precise targeting cytotoxicity of antibody-drug conjugate combined with immunotherapy as first-line regimen for metastatic triple-negative breast cancer in ASCENT-04. Transl Breast Cancer Res. (2025) 6:29. doi: 10.21037/tbcr-25-32, PMID: 40756953 PMC12314681

[37] Tolaney SM DeMichele A Takano T Rugo HS Perou C Lynce F . OptimICE-RD: sacituzumab govitecan + pembrolizumab vs pembrolizumab (± capecitabine) for residual triple-negative breast cancer. Future Oncol. (2024) 20:2343–55. doi: 10.1080/14796694.2024.2357534, PMID: 38922307 PMC11520537

[38] Wang X Collet L Rediti M Debien V De Caluwé A Venet D . Predictive biomarkers for response to immunotherapy in triple negative breast cancer: promises and challenges. J Clin Med. (2023) 12:953. doi: 10.3390/jcm12030953, PMID: 36769602 PMC9917763

[39] Saini KS Punie K Twelves C Bortini S de Azambuja E Anderson S . Antibody-drug conjugates, immune-checkpoint inhibitors, and their combination in breast cancer therapeutics. Expert Opin Biol Ther. (2021) 21:945–62. doi: 10.1080/14712598.2021.1936494, PMID: 34043927

[40] Zhu X Huang Y Liu J Kong B Cui C Han G . Comprehensive metabolomics study identifies SN-38 organ specific toxicity in mice. Sci Rep. (2025) 15:16405. doi: 10.1038/s41598-025-01753-1, PMID: 40355563 PMC12069665

[41] Turner N Dent RA O'Shaughnessy J Kim SB Isakoff SJ Barrios C . Ipatasertib plus paclitaxel for PIK3CA/AKT1/PTEN-altered hormone receptor-positive HER2-negative advanced breast cancer: primary results from cohort B of the IPATunity130 randomized phase 3 trial. Breast Cancer Res Treat. (2022) 191:565–76. doi: 10.1007/s10549-021-06450-x, PMID: 34860318 PMC8831286

[42] Manna M Brabant M Greene R Chamberlain MD Kumar A Alimohamed N . Canadian expert recommendations on safety overview and toxicity management strategies for sacituzumab govitecan based on use in metastatic triple-negative breast cancer. Curr Oncol. (2024) 31:5694–708. doi: 10.3390/curroncol31090422, PMID: 39330050 PMC11431578

[43] Spring LM Nakajima E Hutchinson J Viscosi E Blouin G Weekes C . Sacituzumab govitecan for metastatic triple-negative breast cancer: clinical overview and management of potential toxicities. Oncologist. (2021) 26:827–34. doi: 10.1002/onco.13878, PMID: 34176192 PMC8488774

[44] Pérez-García JM Gion M Ruiz-Borrego M Blancas I López-Miranda E Blanch S . Prevention of sacituzumab govitecan-related neutropenia and diarrhea in patients with HER2-negative advanced breast cancer (PRIMED): an open-label, single-arm, phase 2 trial. EClinicalMedicine. (2025) 85:103309. doi: 10.1016/j.eclinm.2025.103309, PMID: 40606525 PMC12221278

[45] Bardia A Hurvitz SA Tolaney SM Loirat D Punie K Oliveira M . Sacituzumab govitecan in metastatic triple-negative breast cancer. N Engl J Med. (2021) 384:1529–41. doi: 10.1056/NEJMoa2028485, PMID: 33882206

[46] Rugo HS Bardia A Marmé F Cortes J Schmid P Loirat D . Sacituzumab govitecan in hormone receptor-positive/human epidermal growth factor receptor 2-negative metastatic breast cancer. J Clin Oncol. (2022) 40:3365–76. doi: 10.1200/jco.22.01002, PMID: 36027558

[47] Dacoregio MI Michelon I Ernesto do Rego Castro C Cezar Aquino de Moraes F Rossato de Almeida G Ravani LV . Safety profile of sacituzumab govitecan in patients with breast cancer: A systematic review and meta-analysis. Breast. (2025) 79:103853. doi: 10.1016/j.breast.2024.103853, PMID: 39616817 PMC11648803

[48] Li X Zhang L Hu S Liu D Hu B Ran J . Postmarketing safety of sacituzumab govitecan: A pharmacovigilance study based on the FDA adverse event reporting system. Clin Pharmacol Ther. (2024) 115:256–68. doi: 10.1002/cpt.3098, PMID: 37994531

[49] Sathe AG Diderichsen PM Fauchet F Phan SC Girish S Othman AA . Exposure-response analyses of sacituzumab govitecan efficacy and safety in patients with metastatic triple-negative breast cancer. Clin Pharmacol Ther. (2025) 117:570–78. doi: 10.1002/cpt.3495, PMID: 39543869 PMC11739744

[50] Qiu S Zhang J Wang Z Lan H Hou J Zhang N . Targeting Trop-2 in cancer: Recent research progress and clinical application. Biochim Biophys Acta Rev Cancer. (2023) 1878:188902. doi: 10.1016/j.bbcan.2023.188902, PMID: 37121444

[51] Paulík A Nekvindová J Filip S . Irinotecan toxicity during treatment of metastatic colorectal cancer: focus on pharmacogenomics and personalized medicine. Tumori. (2020) 106:87–94. doi: 10.1177/0300891618811283, PMID: 30514181

[52] Cai X Tian C Wang L Zhuang R Zhang X Guo Y . Correlative analysis of plasma SN-38 levels and DPD activity with outcomes of FOLFIRI regimen for metastatic colorectal cancer with UGT1A1 *28 and *6 wild type and its implication for individualized chemotherapy. Cancer Biol Ther. (2017) 18:186–93. doi: 10.1080/15384047.2017.1294286, PMID: 28278081 PMC5389420

[53] Crowley F Sheppard R Lehrman S Easton E Marron TU Doroshow D . Optimizing care in early phase cancer trials: The role of palliative care. Cancer Treat Rev. (2024) 128:102767. doi: 10.1016/j.ctrv.2024.102767, PMID: 38776612

[54] Cabibbo G Aghemo A Lai Q Masarone M Montagnese S Ponziani FR . Optimizing systemic therapy for advanced hepatocellular carcinoma: the key role of liver function. Dig Liver Dis. (2022) 54:452–60. doi: 10.1016/j.dld.2022.01.122, PMID: 35131176

[55] Zhang Y Chen J Wang X Wang H Chen X Hong J . Efficacy and safety of Sacituzumab govitecan in solid tumors: a systematic review and meta-analysis. Front Oncol. (2025) 15:1624386. doi: 10.3389/fonc.2025.1624386, PMID: 40626021 PMC12231463

[56] Gluz O Xu B Nanda R Dasgupta A Kaushik A Verret W . Efficacy of sacituzumab govitecan versus treatment of physician's choice in previously treated HR+ and HER2- mBC: a meta-analysis of TROPiCS-02 and EVER-132–002 trials. Ther Adv Med Oncol. (2025) 17:17588359251320285. doi: 10.1177/17588359251320285, PMID: 40093981 PMC11907608

[57] Wong SK Nebhan CA Johnson DB . Impact of patient age on clinical efficacy and toxicity of checkpoint inhibitor therapy. Front Immunol. (2021) 12:786046. doi: 10.3389/fimmu.2021.786046, PMID: 34868071 PMC8635107

[58] Li R Hensley PJ Gupta S Al-Ahmadie H Babjuk M Black PC . Bladder-sparing therapy for bacillus calmette-guérin-unresponsive non-muscle-invasive bladder cancer: international bladder cancer group recommendations for optimal sequencing and patient selection. Eur Urol. (2024) 86:516–27. doi: 10.1016/j.eururo.2024.08.001, PMID: 39183090

[59] Segal JB Varadhan R Groenwold RHH Li X Nomura K Kaplan S . Assessing heterogeneity of treatment effect in real-world data. Ann Intern Med. (2023) 176:536–44. doi: 10.7326/m22-1510, PMID: 36940440 PMC10273137

[60] Loriot Y Balar AV Petrylak DP Kalebasty AR Grivas P Fléchon A . Sacituzumab govitecan demonstrates efficacy across tumor trop-2 expression levels in patients with advanced urothelial cancer. Clin Cancer Res. (2024) 30:3179–88. doi: 10.1158/1078-0432.Ccr-23-3924, PMID: 39086310

[61] Bardia A Rugo HS Cortés J Tolaney SM Schmid P Motwani M . Trop-2 mRNA expression and association with clinical outcomes with sacituzumab govitecan (SG) in patients with HR+/HER2– metastatic breast cancer (mBC): Biomarker results from the phase 3 TROPiCS-02 study. J Clin Oncol. (2023) 41:1082–82. doi: 10.1200/JCO.2023.41.16_suppl.1082

[62] Rugo HS Bardia A Marme F Cortes J Schmid P Loirat D . PS3–2 Final OS analysis from the phase 3 TROPiCS-02 study: sacituzumab govitecan in HR+/HER2&x2212; metastatic breast cancer. Ann Oncol. (2024) 35:S1314–S15. doi: 10.1016/j.annonc.2024.07.716

[63] LeVee A Wong M Ruel N Schmolze D Han M Mortimer J . Trop-2 expression as a biomarker of response to sacituzumab govitecan in patients with HER2-negative metastatic breast cancer: A pilot study. Cancer Treat Res Commun. (2025) 44:100954. doi: 10.1016/j.ctarc.2025.100954, PMID: 40516178

[64] Khoury K Feldman R Pohlmann PR Heeke AL Gatalica Z Veloso Y . Molecular characterization of trophoblast cell surface antigen 2 (Trop-2) positive triple negative breast cancer (TNBC). J Clin Oncol. (2019) 37:e14651–e51. doi: 10.1200/JCO.2019.37.15_suppl.e14651

[65] Hammood M Craig AW Leyton JV . Impact of endocytosis mechanisms for the receptors targeted by the currently approved antibody-drug conjugates (ADCs)-A necessity for future ADC research and development. Pharm (Basel). (2021) 14:674. doi: 10.3390/ph14070674, PMID: 34358100 PMC8308841

[66] Mebrahtu A Laurén I Veerman R Akpinar GG Lord M Kostakis A . A bispecific CD40 agonistic antibody allowing for antibody-peptide conjugate formation to enable cancer-specific peptide delivery, resulting in improved T proliferation and anti-tumor immunity in mice. Nat Commun. (2024) 15:9542. doi: 10.1038/s41467-024-53839-5, PMID: 39500897 PMC11538452

[67] Zhao D Zhao J Li Y Cai X Yang Z Zhou F . Circulating tumor DNA (ctDNA) monitoring in the assessment and prediction of the efficacy of PARP inhibitors (PARPi). J Clin Oncol. (2025) 43:TPS307–TPS07. doi: 10.1200/JCO.2025.43.5_suppl.TPS307

[68] von Arx C De Placido P Caltavituro A Di Rienzo R Buonaiuto R De Laurentiis M . The evolving therapeutic landscape of trastuzumab-drug conjugates: Future perspectives beyond HER2-positive breast cancer. Cancer Treat Rev. (2023) 113:102500. doi: 10.1016/j.ctrv.2022.102500, PMID: 36587473

[69] Pavone G Motta L Martorana F Motta G Vigneri P . A new kid on the block: sacituzumab govitecan for the treatment of breast cancer and other solid tumors. Molecules. (2021) 26:7294. doi: 10.3390/molecules26237294, PMID: 34885875 PMC8659286

[70] Swain SM Nishino M Lancaster LH Li BT Nicholson AG Bartholmai BJ . Multidisciplinary clinical guidance on trastuzumab deruxtecan (T-DXd)-related interstitial lung disease/pneumonitis-Focus on proactive monitoring, diagnosis, and management. Cancer Treat Rev. (2022) 106:102378. doi: 10.1016/j.ctrv.2022.102378, PMID: 35430509

