# Supplementary figures and images for: The efficacy and safety of sacituzumab govitecan in the treatment of breast cancer: a systemic review and meta-analysis of emerging clinical data
Source: Front Immunol. 2025 Nov 6;16:1683594. doi: 10.3389/fimmu.2025.1683594 (PMC12629933; doi:10.3389/fimmu.2025.1683594)

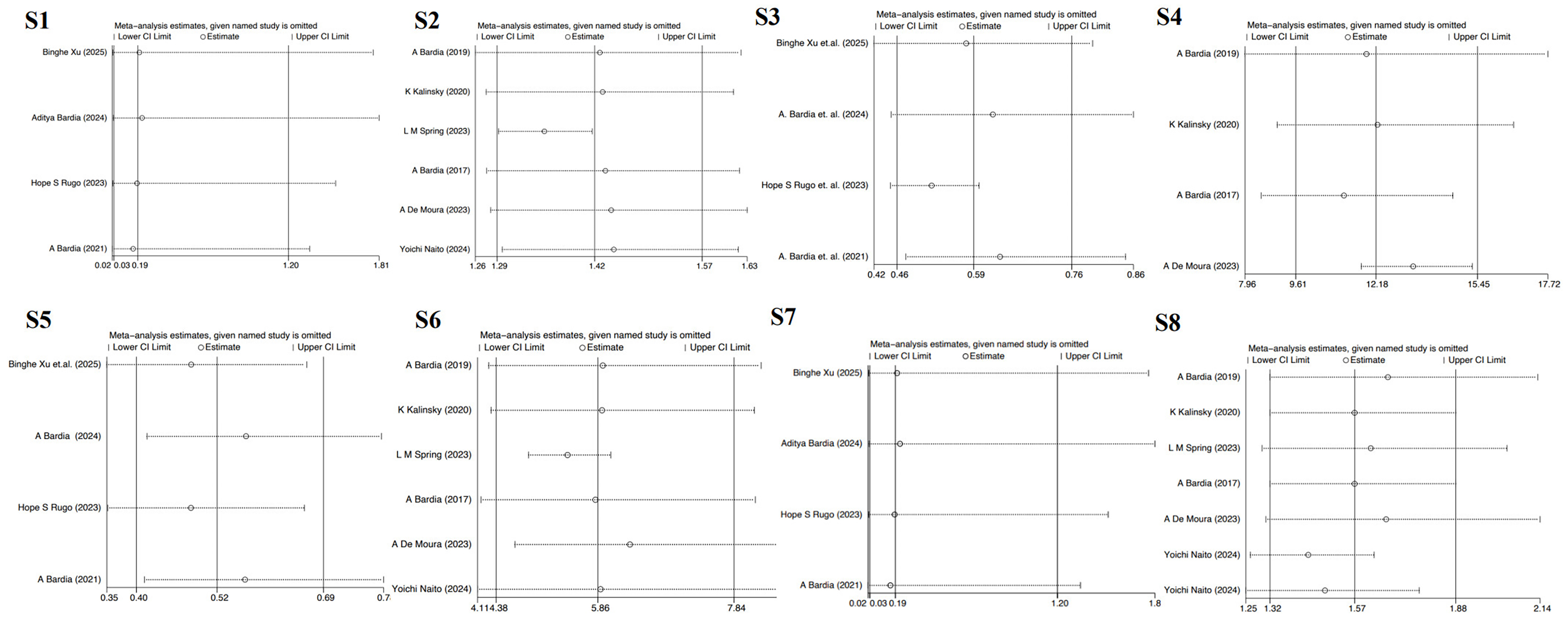

Supplement: Supplementary Figure 1 — Sensitivity analysis of ORR in RCTs by omitting one study at a time. [file Image1.jpg]
