# Supplementary material for: The efficacy and safety of sacituzumab govitecan in the treatment of breast cancer: a systemic review and meta-analysis of emerging clinical data
Source: Front Immunol. 2025 Nov 6;16:1683594. doi: 10.3389/fimmu.2025.1683594 (PMC12629933; doi:10.3389/fimmu.2025.1683594)
Supplement: Supplementary file 2 [file Table1.docx]

**Supplementary Table 1 Search strategy.**

| **Embase** |  |
| --- | --- |
| #1 | 'Breast Neoplasms'/exp |
| #2 | ('Breast Neoplasms' OR 'Breast Cancer' OR 'Breast Carcinoma' OR 'Breast Gland Cancer' OR 'Breast Gland Neoplasm' OR 'Breast Malignancy' OR 'Breast Malignant Neoplasm' OR 'Breast Malignant Tumor' OR 'Breast Neoplasm' OR 'Breast Tumor' OR 'Ca Breast' OR 'Cancer in the Mammary Gland' OR 'Cancer of Breast' OR 'Cancer of the Breast' OR 'Cancer of the Mammary Gland' OR 'Human Mammary Carcinoma' OR 'Human Mammary Neoplasm' OR 'Malignancy of the Breast' OR 'Malignant Breast Neoplasm' OR 'Malignant Breast Tumor' OR 'Malignant Neoplasm of Breast' OR 'Malignant Tumor of Breast' OR 'Mamma Cancer' OR 'Mammary Cancer' OR 'Mammary Gland Cancer' OR 'Mammary Gland Malignancy' OR 'Mammary Malignancy'):ti,ab,kw |
| #3 | ('sacituzumab govitecan' OR 'IMMU-132' OR 'HRS7-SN38' OR 'Trodelvy' OR 'SG' OR 'sacituzumab govitecanhziy'):ti,ab,kw |
| #4 | (#1 OR #2) AND #3 |
| **Pubmed** |  |
| #1 | Breast Neoplasms[MeSH Terms] |
| #2 | "Breast Neoplasms"[Title/Abstract] OR "Breast Cancer"[Title/Abstract] OR "Breast Carcinoma"[Title/Abstract] OR "Breast Gland Cancer"[Title/Abstract] OR "Breast Gland Neoplasm"[Title/Abstract] OR "Breast Malignancy"[Title/Abstract] OR "Breast Malignant Neoplasm"[Title/Abstract] OR "Breast Malignant Tumor"[Title/Abstract] OR "Breast Neoplasm"[Title/Abstract] OR "Breast Tumor"[Title/Abstract] OR "Ca Breast"[Title/Abstract] OR "Cancer in the Mammary Gland"[Title/Abstract] OR "Cancer of Breast"[Title/Abstract] OR "Cancer of the Breast"[Title/Abstract] OR "Cancer of the Mammary Gland"[Title/Abstract] OR "Human Mammary Carcinoma"[Title/Abstract] OR "Human Mammary Neoplasm"[Title/Abstract] OR "Malignancy of the Breast"[Title/Abstract] OR "Malignant Breast Neoplasm"[Title/Abstract] OR "Malignant Breast Tumor"[Title/Abstract] OR "Malignant Neoplasm of Breast"[Title/Abstract] OR "Malignant Tumor of Breast"[Title/Abstract] OR "Mamma Cancer"[Title/Abstract] OR "Mammary Cancer"[Title/Abstract] OR "Mammary Gland Cancer"[Title/Abstract] OR "Mammary Gland Malignancy"[Title/Abstract] OR "Mammary Malignancy"[Title/Abstract] |
| #3 | "sacituzumab govitecan-hziy"[Title/Abstract] OR "SG"[Title/Abstract] OR "sacituzumab govitecan"[Title/Abstract] OR "IMMU-132"[Title/Abstract] OR "hRS7-SN-38"[Title/Abstract] |
| #4 | (#1 OR #2) AND #3 |
| **The Cochrane library** |  |
| #1 | 'Breast Neoplasms'/exp |
| #2 | ('Breast Neoplasms' OR 'Breast Cancer' OR 'Breast Carcinoma' OR 'Breast Gland Cancer' OR 'Breast Gland Neoplasm' OR 'Breast Malignancy' OR 'Breast Malignant Neoplasm' OR 'Breast Malignant Tumor' OR 'Breast Neoplasm' OR 'Breast Tumor' OR 'Ca Breast' OR 'Cancer in the Mammary Gland' OR 'Cancer of Breast' OR 'Cancer of the Breast' OR 'Cancer of the Mammary Gland' OR 'Human Mammary Carcinoma' OR 'Human Mammary Neoplasm' OR 'Malignancy of the Breast' OR 'Malignant Breast Neoplasm' OR 'Malignant Breast Tumor' OR 'Malignant Neoplasm of Breast' OR 'Malignant Tumor of Breast' OR 'Mamma Cancer' OR 'Mammary Cancer' OR 'Mammary Gland Cancer' OR 'Mammary Gland Malignancy' OR 'Mammary Malignancy'):ti,ab,kw |
| #3 | ('sacituzumab govitecan' OR 'IMMU-132' OR 'HRS7-SN38' OR 'Trodelvy' OR 'SG' OR 'sacituzumab govitecanhziy'):ti,ab,kw |
| #4 | (#1 OR #2) AND #3 |
| **Web of science** |  |
| #1 | TS=(Breast Neoplasms) OR MH=(Breast Neoplasms) |
| #2 | TS=("Breast Cancer" OR "Breast Carcinoma" OR "Breast Gland Cancer" OR "Breast Gland Neoplasm" OR "Breast Malignancy" OR "Breast Malignant Neoplasm" OR "Breast Malignant Tumor" OR "Breast Neoplasm" OR "Breast Tumor" OR "Ca Breast" OR "Cancer in the Mammary Gland" OR "Cancer of Breast" OR "Cancer of the Breast" OR "Cancer of the Mammary Gland" OR "Human Mammary Carcinoma" OR "Human Mammary Neoplasm" OR "Malignancy of the Breast" OR "Malignant Breast Neoplasm" OR "Malignant Breast Tumor" OR "Malignant Neoplasm of Breast" OR "Malignant Tumor of Breast" OR "Mamma Cancer" OR "Mammary Cancer" OR "Mammary Gland Cancer" OR "Mammary Gland Malignancy" OR "Mammary Malignancy") |
| #3 | TS=("sacituzumab govitecan" OR "IMMU-132" OR "HRS7-SN38" OR "Trodelvy" OR "SG" OR "sacituzumab govitecanhziy") |
| #4 | (#1 OR #2) AND #3 |
